# STING agonists reduce genital chlamydial infection and attenuate inflammation and pathology

**DOI:** 10.1128/iai.00761-25

**Published:** 2026-04-30

**Authors:** Xuemei Wang, Cuixiu Wu, Yidan Gao, Lijuan Jiang, Yuanshuo Guo, Na Cui, Qian Wang, Haoneng Tang, Shuping Hou, Chunfu Yang, Lingli Tang

**Affiliations:** 1 Experimental Center, Guizhou Center for Disease Control and Prevention 577390, Guiyang, China; 2 Department of Laboratory Medicine, The Second Xiangya Hospital of Central South University 70566, Changsha, China; 3 Department of Dermatovenereology, Tianjin Medical University General Hospital/Tianjin Institute of Sexually Transmitted Disease, Tianjin, China; 4 School of Public Health and Emergency Management, School of Medicine, Southern University of Science and Technology 255310, Shenzhen, China; University of Virginia, Charlottesville, Virginia, USA

**Keywords:** STING agonist, *Chlamydia*, genital infection, 2’3’-cGAMP, diABZI

## Abstract

The cGAS-STING pathway is activated during chlamydial infection and plays a critical role in controlling *Chlamydia trachomatis* infection in the mouse lower genital tract. The current study evaluated whether intravaginal administration of exogenous STING agonists could inhibit chlamydial infection and attenuate upper genital tract pathologies in a mouse model of *Chlamydia muridarum* infection. We found that both the STING agonists 2′3′-cGAMP and diABZI significantly reduced the shedding of live chlamydial organisms recovered from the vaginal swabs and markedly decreased hydrosalpinx and inflammatory infiltrates induced by *C. muridarum*, which are major pathological outcomes associated with tubal infertility in women infected with *C. trachomatis*. Importantly, mice exhibited local and systemic tolerance to the STING agonists. Collectively, these findings suggest a novel STING agonist-based approach for controlling chlamydial infection, which could offer a non-antibiotic, mucosally targeted strategy to reduce the risk of hydrosalpinx in exposed women.

## INTRODUCTION

*Chlamydia trachomatis* infection in the genital tract is a major cause of sexually transmitted disease (1). The control of chlamydial infection is a public-health priority because it may lead to sequelae in women, including chronic pelvic pain, ectopic pregnancy, and infertility (2). Current strategies for controlling *C. trachomatis* infection focus on (i) systematic screening and treatment, (ii) surveillance of antimicrobial resistance (3), and (iii) the development and implementation of vaccines. First, pharmacological activation of mucosal innate immune responses, with consequent amplification of cellular immunity, can synergize with conventional antibiotics or antigen-specific vaccines to achieve more effective bactericidal kinetics while simultaneously attenuating infection-associated immunopathology (4). Additionally, given the complexity of controlling *C. trachomatis,* vaccine development and implementation have emerged as the most promising and cost-effective intervention (5). In line with this, the World Health Organization has identified the development of an effective *C. trachomatis* vaccine as a research priority in the field of sexually transmitted infections (6). Ideally, a *C. trachomatis* vaccine should not only prevent infection but also reduce the burden of upper genital tract sequelae in women. To achieve these goals, the vaccine should potently activate the host’s innate immune system, thereby eliciting a high-quality mucosal adaptive response that ensures early defense against chlamydial infection and long-term host protection (7).

STING agonists are emerging activators of innate immunity that engage the cGAS-STING pathway and subsequently shape cellular and adaptive immune responses (8). To date, their applications have primarily focused on oncology, anti-infective therapy, and vaccine adjuvant (9–11). STING agonists can be broadly divided into cyclic dinucleotide (CDN) and non-CDN families (12). The canonical natural CDN agonist, 2′3′-cGAMP, potently triggers STING-dependent innate signaling and protects against microbial invasion, fulfilling key criteria for a next-generation vaccine adjuvant. It can be rapidly hydrolyzed by ectonucleotide pyrophosphatase 1, limiting systemic exposure and STING activation, thereby minimizing the risk of systemic inflammation (13), and alleviating mucosal epithelial tissue damage during cancer chemotherapy (14). When formulated as an intranasal spray with inactivated influenza virus, 2′3′-cGAMP elicits robust heterosubtypic immunity in mice and ferrets, conferring broad protection against five influenza strains (15). diABZI is a potent non-nucleotide STING agonist whose physicochemical properties differ from those of classic CDNs, affording superior bioavailability. Both *in vitro* and *in vivo*, it preferentially induces type I interferon responses with modest pro-inflammatory cytokine production, with IFN-β predominating *in vivo* (16). diABZI has been shown to suppress infection by multiple SARS-CoV-2 variants (17), influenza viruses, and parainfluenza viruses through its potent interferon response (18). By acting on antigen-presenting cells such as dendritic cells (19) and macrophages (20, 21), these agonists not only trigger the STING-type I interferon signaling axis but also enhance T-cell immunity and promote robust neutralizing antibody responses. They have demonstrated strong innate immunostimulatory and antitumor efficacy in multiple preclinical and clinical studies (22). Consequently, their ability to activate innate immunity has driven increasing interest in their therapeutic potential against infectious diseases (23–25) and in vaccine strategies (26–28).

Historically, the use of Toll-like receptor (TLR) agonists to mimic pathogen-associated molecular patterns and thereby reshape the local innate-adaptive immune axis, enhance IFN-γ-dependent clearance, shorten infection duration, and attenuate pathology, has been a central focus of *C. trachomatis* combination therapy and vaccine research (29). However, recent intravaginal studies have shown that STING agonists trigger faster and more robust interferon responses than do TLR agonists, whereas TLR agonists provoke markedly higher levels of pro-inflammatory cytokines such as TNF-α (24). These findings suggest that STING agonists may offer improved efficacy and safety. The protective function of the cGAS-STING pathway during chlamydial challenge (30), together with the more severe salpingitis observed in STING-deficient mice (31), strongly suggests that activating this pathway is beneficial for both infection control and tissue protection. Chlamydial cyclic di-AMP, a nucleotide metabolite, binds STING and rapidly induces type I interferon response, IL-1β, and IL-18, implying an intrinsic natural adjuvant effect (32). We therefore hypothesize that, beyond their antiviral activity, STING agonists may enhance anti-*Chlamydia* immunity with a superior safety-efficacy profile compared to TLR agonists, as evidenced by recent studies showing their efficacy in cancer treatment and against SARS-CoV-2. At present, evidence supporting the use of STING agonists in chlamydial infection remains limited. Only recently, Poston and colleagues combined the chlamydial protease-like activity factor with the STING agonist ADU-S100 as a vaccine; intranasal administration efficiently reduced *Chlamydia muridarum* infection in the genital tract (33). Whether direct application of STING agonists to the vaginal mucosa can protect against *C. muridarum* infection and alleviate subsequent pathology has not been explored. Here, we demonstrate that two distinct STING agonists, 2′3′-cGAMP and diABZI, each confer significant protection against *C. muridarum* infection and, importantly, reduce hydrosalpinx and inflammatory infiltrates. Harnessing innate immunity through STING activation may therefore represent a promising therapeutic strategy for exposed women.

## MATERIALS AND METHODS

### Chlamydial organism growth

The *C. muridarum* organisms used in the current study were derived from strain Nigg3 (GenBank accession number: CP009760.1). The organisms were propagated in HeLa cells (human cervical carcinoma epithelial cells; ATCC CCL-2), and elementary bodies (EBs) were purified as reported previously (34). Aliquots of purified EBs were stored at −80°C until use. The storage buffer was sucrose-phosphate-glutamate (SPG), consisting of 218 mM sucrose, 10.8 mM phosphate, and 5.6 mM L-glutamic acid, pH 7.2.

### Mouse infection

All mice used in the current study were 6–8-week-old female C57BL/6J mice and were purchased from Hunan Slake Jingda Laboratory Animals Co., Ltd. Each mouse was inoculated intravaginally with 2 × 10^5^ inclusion-forming units (IFUs) of *C. muridarum* EBs as described previously (35). Briefly, stock EBs diluted in 10 μL SPG that contained 2 × 10^5^ IFUs were delivered to the ectocervix area using a 20 μL micropipette tip. Five days prior to inoculation, each mouse was injected subcutaneously with 2.5 mg medroxyprogesterone (Depo-Provera; Abcam, USA) suspended in sterile phosphate-buffered saline (PBS). Following inoculation, mice were monitored for live organism shedding using vaginal swabs. On day 56 after intravaginal inoculation, all mice were sacrificed for blood collecting and evaluating of the genital tract pathology as described below.

**Fig 1:**
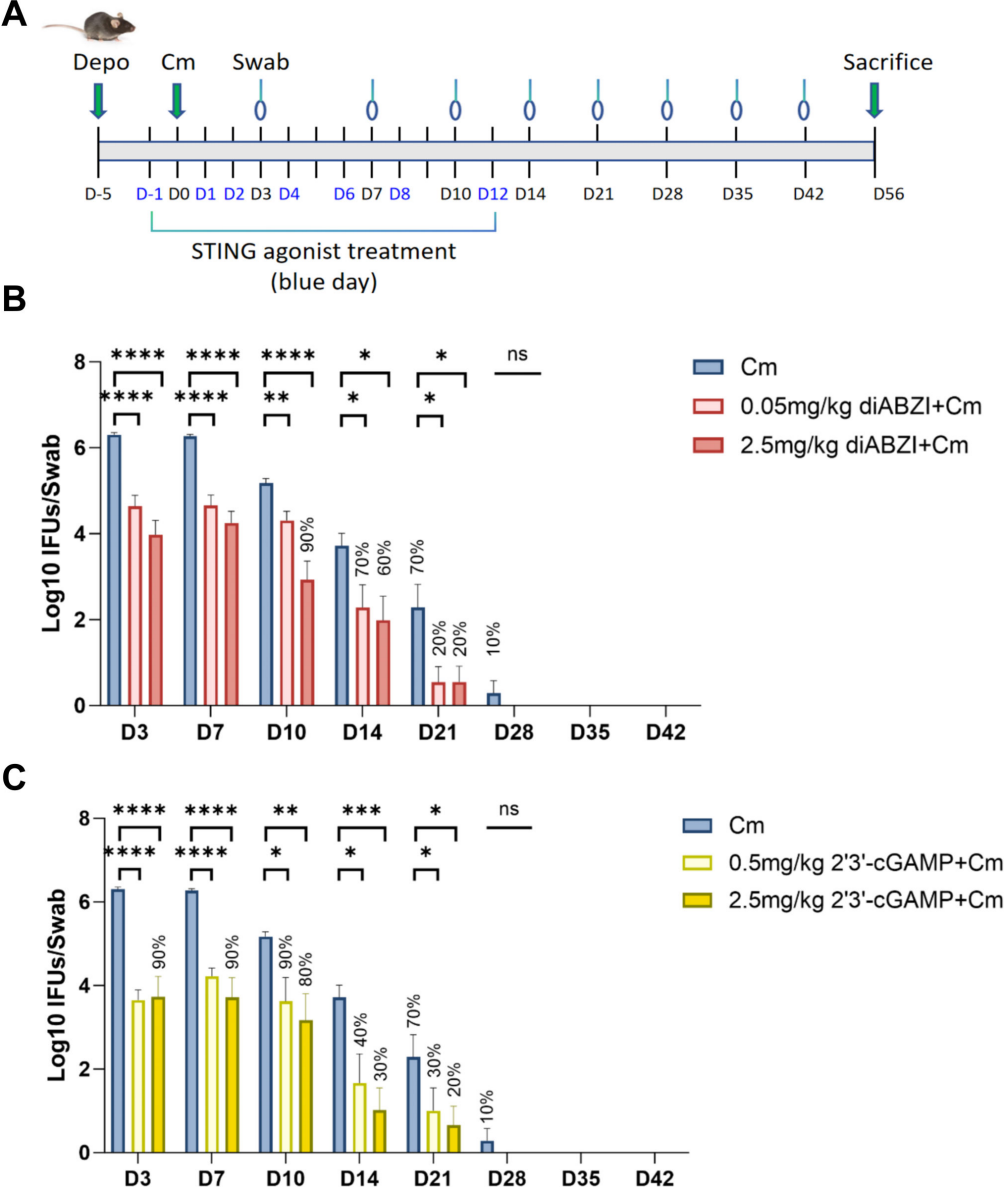
diABZI and 2′3′-cGAMP significantly reduced the chlamydial burdens following a lower genital tract infection. (**A**) Illustration of the timeline for the treatment regimens. Mice were divided into different treatment groups and intravaginally administered PBS (with 2% DMSO), 0.05 mg/kg diABZI, or 2.5 mg/kg diABZI (dissolved in 2% DMSO), 0.5 mg/kg 2′3′-cGAMP, or 2.5 mg/kg 2′3′-cGAMP (dissolved in PBS), respectively, followed by intravaginal challenge with *C. muridarum* (2 × 10^5^ IFUs/mouse) at the designated time points. (**B and C**) The log10 IFUs of live organisms per swab at indicated time points after treatment with diABZI (**B**) or 2′3′-cGAMP (**C**). The percentages of mice with positive detection of chlamydial organisms in swab samples are indicated on top of the bars when the percentages in the corresponding groups are less than 100%. All data at each time point were subjected to ANOVA (all groups) and Wilcoxon rank sum (each dose); error bars = SEM, ns means no significance, *P < 0.05, **P < 0.01, ***P < 0.001, ****P < 0.0001, compared with the *C. muridarum* control group. Data were from two independent experiments. *N* = 10.

Mice were treated with STING agonists diABZI (catalog no. HY-112921B; MedChemExpress, USA) or 2′3′-cGAMP (catalog no. HY-100564; MedChemExpress, USA) as indicated in the figures. Briefly, STING agonist (diABZI and 2′3′-cGAMP) was applied to mice 1 day prior to chlamydial infection and at other designated time points, as illustrated in Fig. 1A, and mice were treated intravaginally with 10 μL of the corresponding drug solution using a 20 μL micropipette tip. The drug solution preparation was conducted in accordance with the reagent instructions. The dosages of diABZI and 2′3′-cGAMP used for treating mice were as follows: 0.05 mg/kg diABZI, 2.5 mg/kg diABZI, 0.5 mg/kg 2′3′-cGAMP, and 2.5 mg/kg 2′3′-cGAMP.

### Titrating live chlamydial organisms recovered from mouse vaginal swabs

To monitor live organism shedding from the genital tract, vaginal swabs were taken on days 3, 5, 7, and 10 after intravaginal infection and weekly thereafter until two consecutive negative detection results were obtained from the same mouse. Each swab was soaked in 0.5 mL SPG and vortexed with glass beads to release infectious EBs, which were then inoculated onto HeLa cell monolayers in duplicate. An immunofluorescence assay was used to quantify live organisms; the total number of IFU/swab was converted into a log10 value for calculating the group mean and standard deviation at each time point, as previously described (36).

### Immunofluorescence assay

An immunofluorescence assay was used for titrating live organisms from mouse swab samples. Briefly, HeLa cells cultured in 24-well plates were fixed with 4% paraformaldehyde (Beyotime) and permeabilized with Triton X-100 (Beyotime). After washing and blocking, the cell samples were subjected to a combination of antibody and chemical staining. Mouse serum-derived polyclonal anti-Cm antibody plus a goat anti-mouse IgG conjugated with FITC antibody (green; Proteintech) were used to visualize chlamydial inclusions. Hoechst (blue; Beyotime) was used to visualize nuclear DNA. The immunolabeled cultures were used for counting inclusions under fluorescence microscopy (ZEN).

### Evaluating mouse genital tract pathology macroscopically and microscopically

Mice were euthanized on day 56 after infection to assess genital tract pathology. The genital tract tissues were isolated and grossly examined for oviduct hydrosalpinx or related abnormalities. Hydrosalpinx was visually scored according to an ordinal scale as described previously (37). Subsequently, the tissues were sectioned and subjected to hematoxylin and eosin (H&E) staining to evaluate the histopathology of genital tract inflammation in mice, focusing on oviduct inflammation and uterine distension. As described previously (35), inflammatory cell infiltrates were scored as follows: 0, no significant infiltration; 1, infiltration at a single focus; 2, infiltration at two to four foci; 3, infiltration at more than four foci; and 4, confluent infiltration. Scores from both oviducts in each mouse were combined as the total pathology score, and assigned to individual mice were calculated into means ± standard errors for each group of animals. Scoring for dilatation of the uterus: the vertical distance from one myometrial border to the opposite border and the corresponding luminal width were measured on images captured with Olympus cellSens software. For each specimen, three equally spaced positions were evaluated and averaged, then the mean values from the two horns were combined to yield the total uterine dilation score.

### Enzyme-linked immunosorbent assay

Mouse serum samples were collected, and *C. muridarum*-specific IgG, IgG1, IgG2a, and IgA responses were determined by standard enzyme-linked immunosorbent assay (ELISA). Nunc MaxiSorp 96-well ELISA plates (BioLegend) were coated with 50 µL of a solution containing 1 × 10^4^ activated *C. muridarum* EBs in 50 mM carbonate-bicarbonate buffer at 4°C overnight. After five washes with PBS containing 0.05% Tween-20, plates were blocked for 2 hours at room temperature with PBS containing 5% nonfat dry milk. Serum samples diluted 1:20 for IgA detection and 1:800 for IgG, IgG1, and IgG2a detection in PBS were added and incubated for 2 hours at 37°C. Horseradish peroxidase-conjugated goat anti-mouse IgG (Beyotime, 1:250 dilution) and IgA (Thermo Fisher Scientific, 1:2,000 dilution) were then applied for 1 hour at 37°C. Reactions were developed with 3,3′,5,5′-tetramethylbenzidine (Beyotime). After incubation for 5–15 minutes, the reaction was terminated by adding the stop solution (Beyotime). Optical density (OD) was measured at 450 nm, and the results were expressed as raw OD values.

### Flow cytometry

Flow cytometry was used to monitor changes in inflammatory cell populations in genital-tract tissue and thereby assess local inflammation. Mice were infected with *C. muridarum* and administered the corresponding drugs according to the schedule described previously in Fig. 1A. Then, mice were sacrificed on day 3 and day 14 post-infection (p.i.) for blood collection and separation of genital tracts, respectively. Single-cell suspensions were prepared from genital tracts as previously described (38). Cells were surface-stained with anti-CD45, CD11b, Ly6G, Ly6C, MHC-II, F4/80, and CD86, followed by fixation/permeabilization and intracellular staining for Arg1. After staining, cells labeled with the specified antibody combinations were analyzed using a flow cytometer. Data were analyzed with FlowJo v10.9 software.

### Statistical analyses

The numbers of live chlamydial organisms, expressed as IFUs, were compared between the two groups using the Wilcoxon rank-sum test. When more than two groups were included in a given experiment, one-way ANOVA was first performed to determine the overall differences. Uterine horn distension scores between the two groups were compared using the Mann-Whitney *U* test. Gross pathological changes and oviduct inflammation and dilation scores (ordinal data) were analyzed using the non-parametric Kruskal-Wallis test. A two-sided *P* value <0.05 was considered statistically significant.

## RESULTS

### diABZI and 2′3′-cGAMP significantly reduced the chlamydial burdens following a lower genital tract infection

We selected two representative STING agonists: diABZI, a synthetic dimeric aminobenzimidazole belonging to the non-canonical, non-CDN family, and 2′3′-cGAMP, the canonical CDN prototype. Mice received intravaginal administration of low-dose or high-dose diABZI or 2′3′-cGAMP, and the kinetics of *C. muridarum* clearance from the lower genital tract were monitored, as illustrated in Fig. 1A. Beginning at day 3 p.i., vaginal administration of both STING agonists at varying doses significantly reduced the magnitude and duration of *C. muridarum* shedding, particularly during the early phase of infection. Mice treated with diABZI or 2′3′-cGAMP began to clear *C. muridarum* infection after day 10, whereas untreated mice did not begin clearing the infection until after day 21. All control mice cleared *C. muridarum* by day 35, whereas STING agonist-treated mice had eliminated the organism by day 28. Notably, vaginal chlamydial loads did not differ significantly between the low-dose and high-dose treatment groups, and the high-dose regimen did not further reduce the overall pathogen burden (Fig. 1B and C).

### STING agonists significantly attenuate hydrosalpinx and inflammatory infiltration in the genital tract

**Fig 2:**
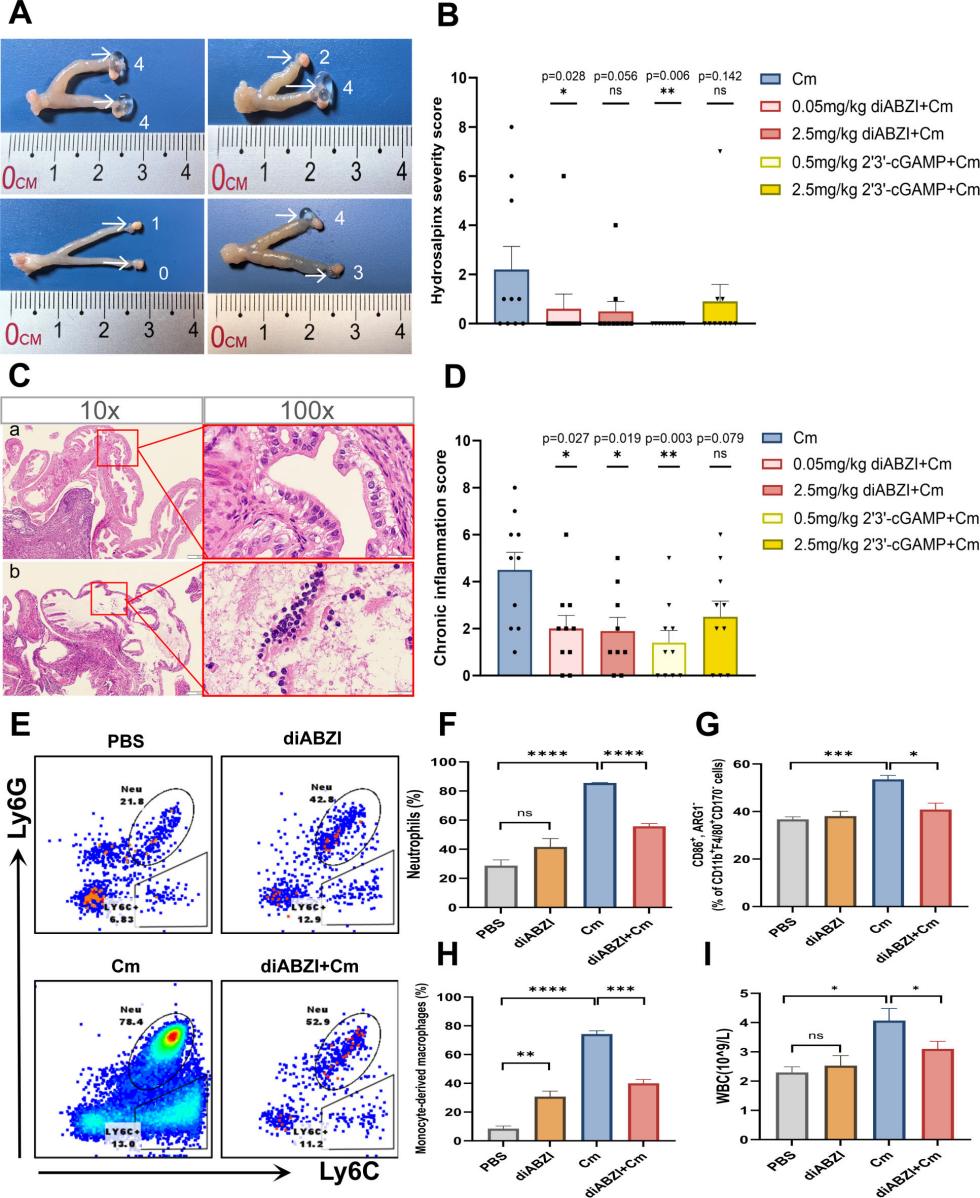
STING agonists significantly attenuate hydrosalpinx and inflammatory infiltration in the genital tract. (**A**) Macroscopic appearance and scoring criteria of the genital tract. (**B**) Total bilateral hydrosalpinx score on day 56 post-infection after treatment with different concentrations of diABZI or 2′3′-cGAMP. (**C**) A representative H&E staining image showing (a) an intact oviduct with normal epithelial architecture and no inflammatory infiltrates and (b) severe inflammatory infiltration and tissue damage in the oviduct. (**D**) Histopathological scores for inflammatory cell infiltration on day 56 post-infection. *N* = 10 mice per group. Non-parametric Kruskal-Wallis rank-sum test was used for ordinal data. Compared with the *C. muridarum* control group, ns means no significance, *P < 0.05, and **P < 0.01. A second set of mice was used for flow cytometric analysis according to the same regimen in Fig. 1A. The treatment group was administered 2.5 mg/kg diABZI, and mice were sacrificed on day 3 and day 14 post-infection. (**E–H**) Flow cytometry analysis of pathogenic pro-inflammatory cells (neutrophils, monocyte-derived macrophages, and M1 type macrophages) in genital tract tissues on day 14 post-infection. (**I**) Peripheral-blood leukocyte counts on day 3 post-infection. *N* = 5 mice per group. Data were analyzed by Mann-Whitney *U* test. *P < 0.05, **P < 0.01, ***P < 0.001, and ****P < 0.0001.

Both diABZI and 2′3′-cGAMP significantly attenuated *Chlamydia*-induced upper genital tract pathology, reducing both the incidence and the severity of hydrosalpinx. In the *C. muridarum* control cohort, hydrosalpinx was observed in 60% of mice. In contrast, only one mouse developed hydrosalpinx in the low-dose diABZI group (0.05 mg kg⁻¹) (10% incidence), and no hydrosalpinx was observed in the low-dose 2′3′-cGAMP group (0.5 mg kg⁻¹). Unexpectedly, high-dose regimens did not abolish genital tract lesions; instead, they modestly increased both the incidence and severity of hydrosalpinx, although the difference did not reach statistical significance. In the 2.5 mg kg⁻¹ diABZI group, two mice (20%) developed hydrosalpinx, whereas three mice (30%) were affected in the equivalent 2.5 mg kg⁻¹ 2′3′-cGAMP group (Fig. 2A and B).

H&E staining revealed oviductal lumina of variable dilation, thinned walls, loss of ciliated epithelium, and inflammatory cell infiltration in *C. muridarum*-infected control mice. In contrast, groups treated with diABZI or 2′3′-cGAMP exhibited significantly reduced inflammatory infiltration and substantially lower chronic inflammation score (Fig. 2C and D). To determine whether acute genital tract inflammation was also alleviated following STING agonist treatment, genital tract tissues were harvested on day 14 p.i., and pro-inflammatory cell populations—primarily neutrophils and monocyte-derived macrophages (mDMs)—were quantified by flow cytometry. diABZI treatment significantly reduced the infiltration of neutrophils, total mDMs, and M1 macrophages into the genital mucosa (Fig. 2E through H), indicating a potential therapeutic effect in reducing inflammatory responses. Moreover, diABZI administration markedly blunted the acute-phase leukocytosis in peripheral blood, indicating its capacity to systemically suppress infection-triggered hyperinflammation (Fig. 2I).

### STING agonists may modulate *Chlamydia*-induced adaptive immune responses

**Fig 3:**
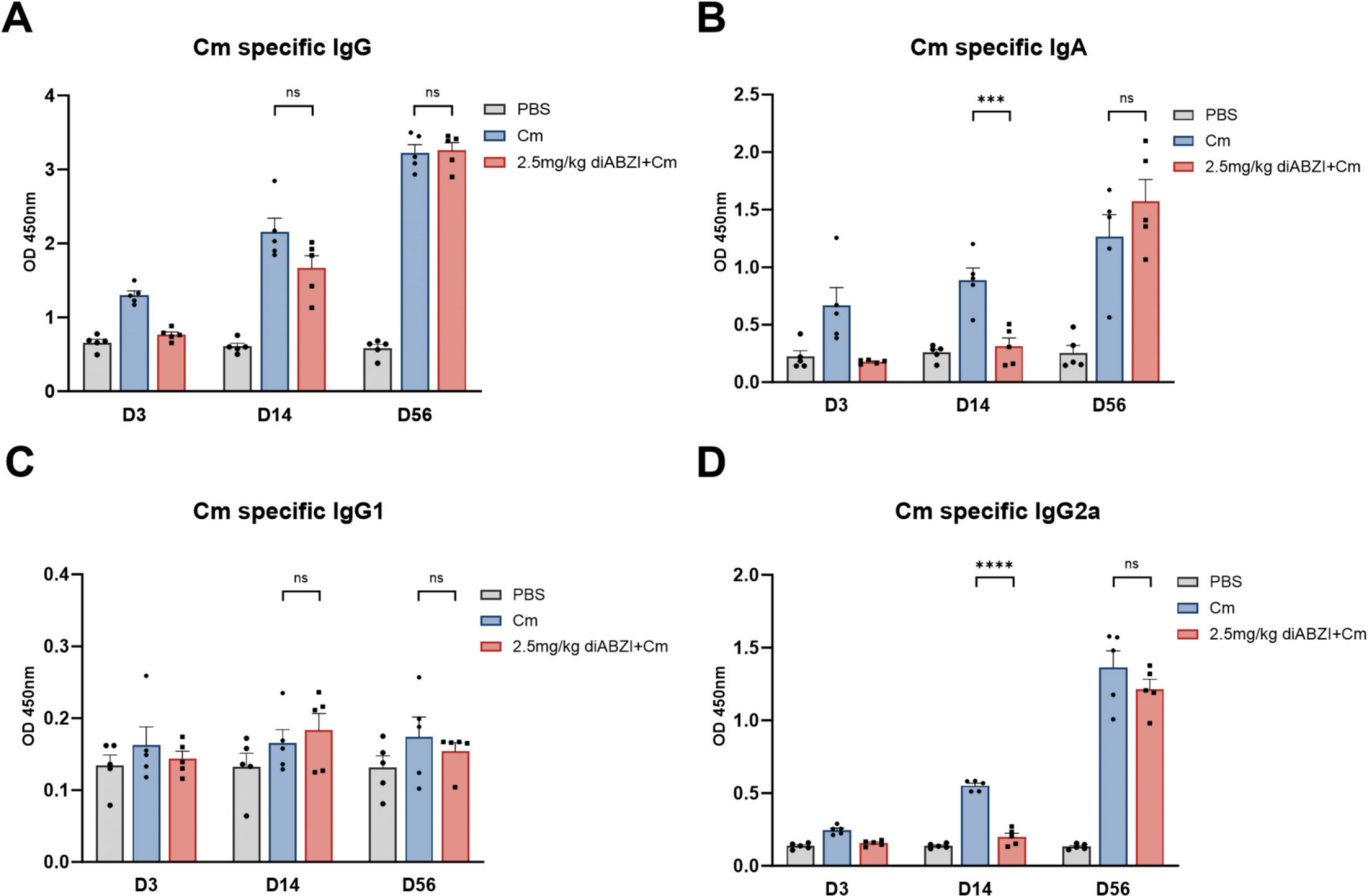
STING agonists may modulate *Chlamydia*-induced adaptive immune response. (**A**) OD 450 nm values of *Chlamydia*-specific IgG, (**B**) IgA, (**C**) IgG1, and (**D**) IgG2a in mouse serum at days 3, 14, and 56 post-infection. Antibody levels were measured by ELISA and expressed as OD 450 nm values. Data are presented as mean ± SEM. Statistical significance was determined by one-way ANOVA with *post hoc* test: **P < 0.01, ***P < 0.001, ****P < 0.0001, and ns means no significance. *N* = 5.

To evaluate the impact of intravaginal administration of STING agonists on adaptive immune responses following chlamydial infection, we monitored the serum levels of *C. muridarum*-specific IgG and its subclasses (IgG1 and IgG2a), as well as IgA, in mouse serum on days 3, 14, and 56 p.i. Compared with day 3 p.i., the OD 450 nm values of IgG, IgG2a, and IgA in both diABZI-treated group and untreated group showed an upward trend on day 14 and day 56 p.i., while IgG1 remained relatively stable. On day 14 p.i., OD 450 nm values of IgG, IgG2a, and IgA in the diABZI-treated group were lower than those in the untreated group (Fig. 3A, B, and D). Notably, on day 56 p.i., no significant differences were observed in the OD 450 nm values of IgG, IgG1, and IgG2a, or IgA between the STING agonist-treated mice and the untreated controls (Fig. 3A through D). These findings indicate that STING agonists may weaken *Chlamydia*-induced humoral immune responses during the early phase of adaptive immunity without impairing long-term antibody responses.

### Intravaginal STING agonists are locally and systemically well tolerated at the intended therapeutic dose and duration

**Fig 4:**
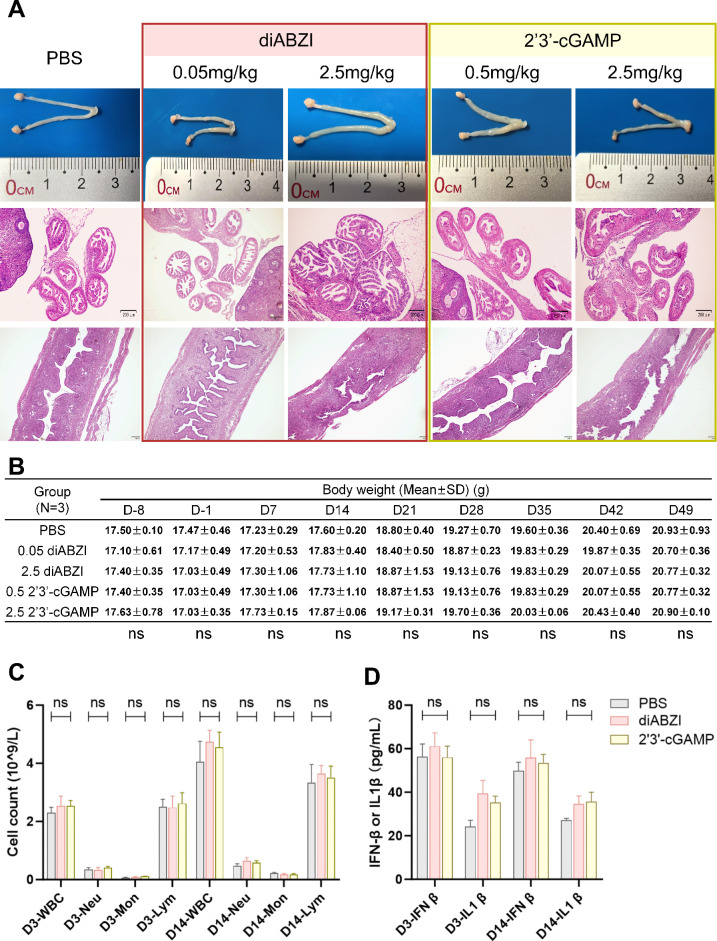
Intravaginal STING agonist is locally and systemically well tolerated at the intended therapeutic dose and duration. (**A**) Macroscopic view of the genital tract (top). Oviduct (mid) and uterus (bottom) morphology in H&E-stained sections on day 56 after treatment with diABZI or 2′3′-cGAMP. (**B**) Body weight of mice in groups; data are presented as mean ± SD, ns indicates no statistically significant difference between groups at any single time point, as determined by one-way ANOVA followed by Dunnett’s post-test (*N* = 3 per group). (**C**) Counts of blood leukocyte subsets and (**D**) serum levels of IFN-β and IL-1β in mice on day 3 and day 14 after treatment with 2.5 mg/kg diABZI or 2′3′-cGAMP. *N* = 5 per group, no significant difference from the control group, as determined by one-way ANOVA followed by Dunnett’s post-test.

Given that STING agonists can elicit dose-limiting inflammatory responses, we first evaluated whether intravaginal administration induces confounding genital tract pathology or body weight changes that might bias subsequent efficacy readouts. To address this, we included a cohort that received the drug only (i.e., without infection) and monitored both local and systemic tolerability. Following exposure to either of two STING agonists, none of the mice exhibited macroscopic genital lesions or histological evidence of inflammatory infiltrates or luminal dilatation in the oviducts or uterus, as assessed by H&E staining (Fig. 4A). During all experiments, including those described above, the body weight of each mouse was monitored regularly, and no abnormal changes were observed, as partially shown in Fig. 4B. In addition, peripheral blood leukocyte subsets and serum levels of IFN-β and IL-1β remained comparable to controls (Fig. 4C and D). Thus, these results indicate that, under the tested dose and duration regimen, intravaginal STING agonists are generally well tolerated and do not induce overt local or systemic inflammation.

### High-dose STING agonist exacerbates uterine pathology following *C. muridarum* infection

**Fig 5:**
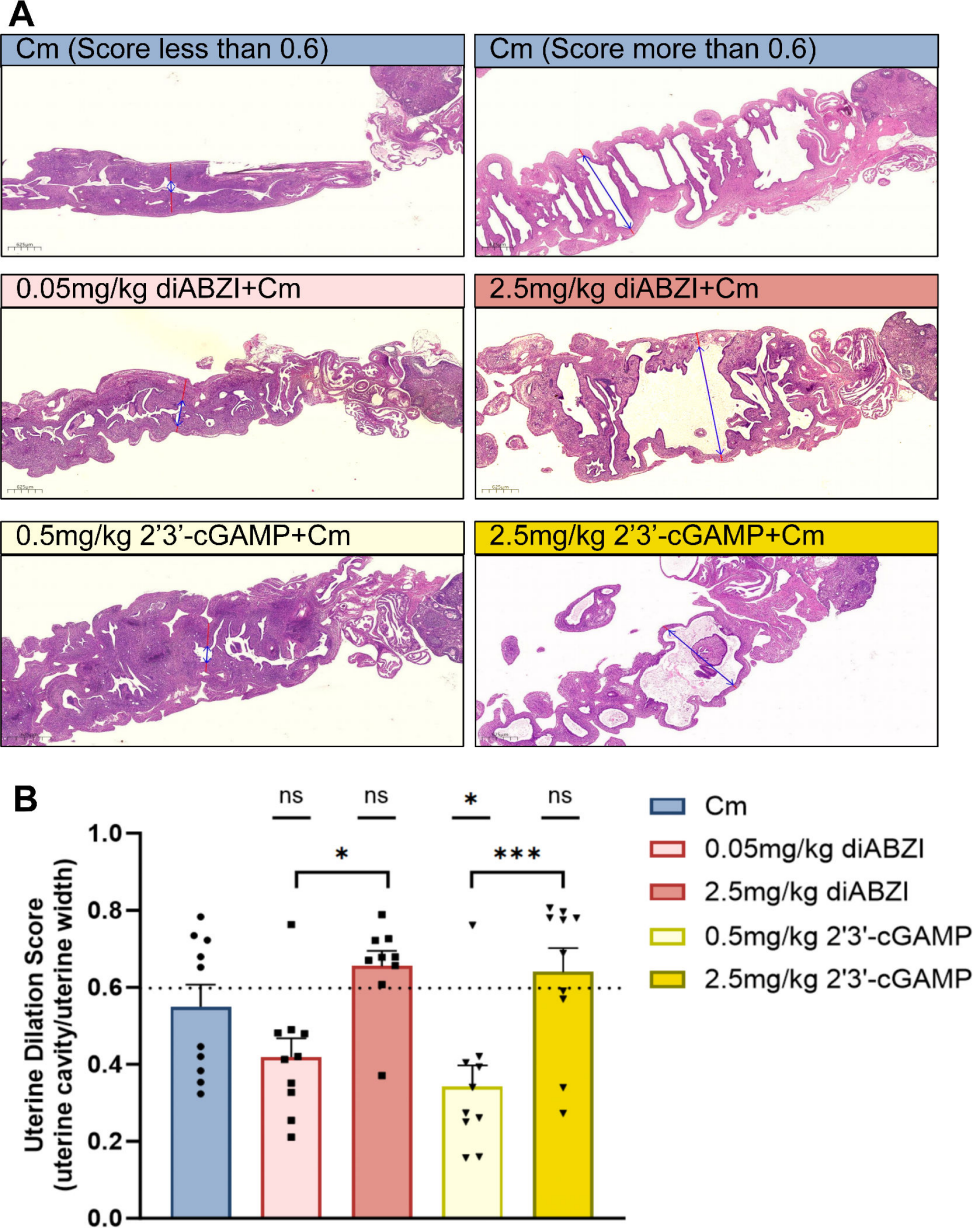
High-dose STING agonist exacerbates uterine pathology following *C. muridarum* infection. (**A**) Schematic representation of a non-distended uterus versus a fluid-distended uterus across groups. In the *C. muridarum*-infected control group, half had distention and half had no distention. (**B**) Uterine distension scores across groups. Short capped lines: comparisons against *Cm*-vehicle control; horizontal brackets: pairwise comparisons. Filled black circles, triangles, and squares indicate individual values from different groups. Data were analyzed using the two-tailed Mann-Whitney *U* test, ns means no significance, *P < 0.05, and ***P < 0.001. *N* = 10.

Although both low-dose and high-dose intravaginal STING agonist treatments reduced vaginal *C. muridarum* burdens and significantly attenuated upper genital tract pathology, increasing the dose did not further enhance bacterial clearance or completely resolve hydrosalpinx or inflammation. Instead, high-dose treatment was associated with a higher incidence of uterine luminal distension and congestion, whereas low-dose treatment resulted in no overt uterine pathology. As shown in Fig. 5A, non-distended uteri displayed intact endometrial architecture with abundant glands and no inflammation, whereas infected distended uteri exhibited luminal dilatation, marked glandular loss, and dense neutrophilic infiltrates. High-dose regimens induced excessive uterine fluid accumulation and inflammatory infiltrates (*P* < 0.01 versus low dose) (Fig. 5A and B). These findings reveal a narrow therapeutic window for intravaginal STING agonists: low doses confer robust protection against infection and pathology, whereas higher doses exacerbate uterine inflammation in a dose-dependent manner.

## DISCUSSION

To evaluate whether intravaginal administration of exogenous STING agonists can inhibit chlamydial infection and attenuate reproductive-tract pathology and inflammation, C57BL/6 mice were intravaginally infected with 2 × 10^5^ IFUs of *C. muridarum*. One day prior to challenge and on each designated day post-infection, mice in the low-dose and high-dose groups received intravaginal diABZI (a non-CDN STING agonist) or 2′3′-cGAMP (a classical CDN STING agonist). Live chlamydial loads in vaginal swabs and reproductive-tract pathology were subsequently assessed.

Both STING agonists significantly reduced chlamydial shedding, which correlated with a marked decrease in neutrophils and pro-inflammatory macrophages—key drivers of *C. muridarum*-induced immunopathology (39, 40)—and was accompanied by reduced hydrosalpinx and chronic inflammatory infiltrates. Notably, low-dose treatment alone significantly accelerated pathogen clearance and ameliorated reproductive-tract pathology. These findings are highly encouraging from both an experimental and a translational perspective, highlighting a clear dose-sparing advantage and confirming potent *in vivo* antimicrobial activity. Moreover, they underscore the critical role of bolstering innate immunity against *C. trachomatis* and providing an experimental framework for novel immunomodulatory strategies. For example, Zhang Conggang and colleagues demonstrated that vitamin D3 promotes transmembrane transport of cyclic 2′3′-GMP-AMP (cGAMP) by inducing endogenous LL-37, thereby robustly activating the STING pathway and enhancing IFN-β mediated antiviral immunity. This finding offers a mechanistic explanation for the long-standing question of how vitamin D exerts antiviral activity (41). In the context of chlamydial infection, vitamin D supplementation has been reported to improve treatment outcomes and may serve as an adjunct to antibiotic therapy (42). Together, these observations prompt the hypothesis that vitamin D status, STING signaling, and clinical outcomes in *C. trachomatis* infection are mechanistically connected. Direct mechanistic experiments were not performed in the present study and remain to be undertaken. Given the pivotal role of the cGAS-STING pathway in defense against *C. trachomatis* infection (30, 31, 43), it is imperative to further explore its therapeutic potential and underlying mechanisms in this context.

Mucosal innate immunity plays a critical role in defending against chlamydial infection (4, 44), while Th1-type cell-mediated immune responses are major contributors to pathogen clearance, characterized by elevated serum IgG2a, but not IgG1 (45). The elevated OD 450 nm values of IgG2a observed post-infection indicate that a Th1-type immune response may have been initiated. Interestingly, the OD 450 nm values of IgG, IgG2a, and IgA in the STING agonist-treated group were lower than those in the control group on day 14 p.i. Although humoral responses in the STING agonist-treated group were modest initially on day 14 p.i., the overall outcome on day 56 p.i. remains favorable, as chronic sequelae of chlamydial infection are primarily driven by persistent infection and inflammation; rapid pathogen clearance is therefore critical. We hypothesized that the STING agonist may activate mucosal innate immunity and protective cell-mediated immunity, thereby suppressing or eliminating most *C. muridarum* infection (46, 47). This rapid pathogen clearance reduces the total antigen load available for recognition by the adaptive immune system (39), thereby delaying or attenuating the initiation of humoral responses. Or perhaps, STING-driven downstream signaling may directly influence B cells by promoting their proliferation, activation, and antibody secretion (10, 48). This could explain why both the STING agonist-treated group and the untreated *C. muridarum* infection group eventually (at day 56 p.i.) developed a comparable level of serum antibody reserves, despite lower initial infection burden and serum antibody levels in the STING agonist-treated group. Collectively, these results indicate that STING agonists could facilitate rapid pathogen clearance via innate or cell-mediated immunity during early infection, thereby reducing inflammatory infiltration and pathological damage. This interpretation is also supported by our histological and flow cytometry data: compared with the single *C. muridarum* infection group, the STING agonist-treated group exhibited significantly reduced chronic inflammation scores at day 56 p.i., as well as decreased pro-inflammatory cells (neutrophils, monocyte-derived macrophages, and M1 macrophages) on day 14 p.i. Notably, monocyte-derived macrophages were markedly increased in the *C. muridarum* infection group and significantly reduced in the STING agonist-treated group, which warrants further investigation. Given that neutrophils express extremely low levels of STING (49), it is worth exploring whether STING agonists exert their antibacterial and pathology-limiting effects primarily through macrophages. Beyond macrophages, the specific target innate immune cells of STING agonists that rapidly respond to chlamydial infection—such as natural killer cells, γδ T cells, and innate lymphoid cell populations present in the reproductive tract—remain to be explored. These cells may play a key role in controlling the early stage of *C. trachomatis* infection by producing innate IFN-γ before the influx of Th1 cells (44, 50). A limitation of this study is that all infected mice sacrificed on day 14 p.i. were used in their entirety to generate single-cell suspensions from genital tract tissues for flow cytometric analysis. Consequently, no residual tissue samples were available for parallel analysis of chlamydial presence/distribution in tissues, and suitable antibodies for flow cytometric detection of chlamydial were not available at the time. These data are important for follow-up studies because, in the absence of tissue burden data, it is difficult to determine whether the inflammatory cells are induced by residual pathogens or simply reflect a reparative state following pathogen clearance. Further mechanistic studies focusing on target cells will require concurrent assessment of chlamydial burden in genital tract tissues.

We also observed that the uterus exhibits limited tolerance to STING agonists; high-dose administration elicited uterine injury, likely attributable to a cytokine-storm-related adverse-event profile. Excessive immune activation can translate into collateral tissue inflammation, an inherent liability of small-molecule STING agonists (18, 51). Considering the limitations of this study, further studies should focus on (i) precision tuning of dose and timing to achieve calibrated innate-immune enhancement and (ii) integration with next-generation nano-delivery systems. STING-agonist-loaded particles (lipid nanoparticles, chitosan, etc.) can maintain mucosal drug levels for prolonged periods after injection or mucosal vaccination without evoking overt histopathology, offering a translational roadmap for mucosal vaccine design (52). Under the present regimen, no appreciable body-weight loss, macroscopic reproductive-tract abnormalities, or immune-cell infiltrates were observed. Although these data argue against acute local irritation or gross toxicity, comprehensive safety endpoints—including systemic biochemistry, reproductive organ indices, and detailed immunophenotyping—are prerequisites before claims of general safety and tolerability can be made. At that point, superior drug design and a more precise dose-time regimen will be required.

The study supports the potential of STING agonists as an anti-infection strategy, particularly in reducing upper reproductive-tract pathology. We suggest a more targeted intravaginal route of immunomodulation. For vaginal infections, topical delivery (gels, suppositories) is preferred because it achieves high local drug concentrations while minimizing systemic exposure and adverse effects (53, 54). Our direct intravaginal delivery, rather than injection, significantly reduced the shedding and ameliorated genital tract pathology, yet failed to augment systemic humoral immunity. Nevertheless, it intimates a potential modulation of cellular and adaptive immune responses. STING agonists can serve as excellent vaccine adjuvants (10, 55, 56). Further investigation is warranted to explore STING agonists as mucosal adjuvants for anti-*Chlamydia* vaccines; a more rational immunization strategy must be established. To sum up, this study aimed to investigate the role of mucosal STING activation in host defense against chlamydial infection. While direct clinical translation is not yet feasible, understanding the role of these pathways may have broader translational implications, including preventing long-term complications such as tubal infertility or exploring as vaccine adjuvants. Additionally, our findings provide insights into the development of immunomodulatory strategies against chlamydial infection, including the potential link between vitamin D and the STING pathway. Given the central role of the cGAS-STING axis in host defense against *C. trachomatis*, systematic investigation of the therapeutic value and mechanism is urgently warranted. Parallel efforts should dissect the STING signaling axis in macrophages to define their unresolved dual roles in protection versus pathology, whereas neutrophils, which lack the STING pathway and have been verified in driving immunopathology following chlamydial infection, are not the focus.

### Conclusion

This study is the first to establish an intravaginal STING-agonist delivery regimen to comparatively evaluate two chemically distinct agonists: diABZI and 2′3′-cGAMP, for their impact on *C. muridarum* infection and inflammatory pathology. Vaginal administration of either agonist conferred significant protection against chlamydial infection, significantly attenuated inflammation and chronic pathology, showing a clear dose-dependent efficacy, high potency, and excellent mucosal tolerability. These findings provide experimental proof-of-concept for adopting STING agonists as a non-antibiotic, mucosally targeted strategy to reduce the risk of hydrosalpinx in exposed women.

## Data Availability

The datasets generated during and/or analyzed during the current study are available from the corresponding author on reasonable request.
